# Hyaluronan in the pathogenesis of lung fibrosis associated to autoimmune pulmonary alveolar proteinosis (aPAP)

**DOI:** 10.3389/fmed.2025.1650830

**Published:** 2025-09-03

**Authors:** Raffaele Gentile, Vincenzo Alfredo Marando, Francesca Mariani, Sara Lettieri, Matteo Della Zoppa, Michele Zorzetto, Laura Maria Ciardelli, Riccardo Albertini, Annalisa De Silvestri, Angelo Guido Corsico, Ilaria Campo

**Affiliations:** ^1^Laboratory of Clinical Chemistry, IRCCS Policlinico San Matteo Foundation, Pavia, Italy; ^2^Pneumology Unit, IRCCS Policlinico San Matteo Foundation, Pavia, Italy; ^3^Department of Internal Medicine and Therapeutics, University of Pavia, Pavia, Italy; ^4^Clinical Epidemiology and Biometric Unit, IRCCS Policlinico San Matteo Foundation, Pavia, Italy

**Keywords:** PAP, lung fibrosis, ELF, hyaluronan, serum biomarkers

## Abstract

**Introduction:**

Pulmonary fibrosis is a rare event occurring in patients with autoimmune Pulmonary Alveolar Proteinosis (aPAP). The accumulation of intra-alveolar material may promote proinflammatory and profibrotic pathways.

**Aim:**

To investigate serum biomarkers as prognostic factor of lung fibrosis in aPAP.

**Methods:**

We performed the ELF™ test (Siemens), which provides a score of fibrosis based on quantitative measurements of hyaluronic acid (HA), amino-terminal propeptide of type III procollagen (PIIINP), and tissue inhibitor of metalloproteinase 1 (TIMP-1) from serum of 21 aPAP patients, collected at diagnosis.

**Results:**

In this retrospective cohort study, we analyzed the serum samples collected at aPAP diagnosis from10 patients (PAP-FIB) showing lung fibrosis evidence on chest HRTC and 11 patients (PAP) who did not develop lung fibrosis within at least 9 years from aPAP diagnosis. Both PAP-FIB and PAP groups exhibited ELF scores above the risk threshold (>7.7), with significantly higher values in PAP-FIB (mean ELF score: 9.19 ± 0.75 vs. 8.52 ± 0.46, *p* = 0.02). Mean HA levels were also significantly elevated in PAP-FIB compared to PAP (55.61 ± 42.01 ng/mL vs. 22.83 ± 9.13 ng/mL, *p* = 0.02). After adjusting for differences in DLCO, HA remained a marginally significant predictor of fibrosis (*p* = 0.05) while the difference in ELF scores between the two groups was no longer statistically significant. ROC analysis demonstrated an AUROC of 0.8, with an optimal cut-off of 9 (sensitivity 70%, specificity 91%) for ELF and an AUROC of 0.8, with a cut-off of 41 ng/mL (100% specificity and 60% sensitivity) for HA.

**Conclusion:**

Our results seem to indicate that a robust HA production contributes to the chronic inflammatory and micro-injury to the alveolar epithelium. The high specificity of HA highlights its utility as a prognostic biomarker of aPAP-associated fibrosis.

## 1 Introduction

Pulmonary Alveolar Proteinosis (PAP) is a rare lung syndrome [with a prevalence of approximately 7 cases per million in the general population (1)] characterized by the accumulation of surfactant lipids and proteins within the alveoli, leading to impaired gas exchange and progressive respiratory insufficiency. PAP can be classified into three main types: primary (caused by disruption of granulocyte-macrophage colony-stimulating factor, GM-CSF, signaling), secondary (due to underlying conditions affecting macrophage function), and congenital (linked to genetic mutations affecting surfactant metabolism) (2). While some patients experience stable or slowly progressive disease, others develop lung fibrosis, a severe complication associated with irreversible tissue remodeling, reduced lung compliance, and worsening respiratory function (3).

Pulmonary fibrosis (PF) is driven by ongoing microscopic damage to the alveolar epithelium, which triggers an abnormal repair process and leads to epithelial dysfunction. This defective healing response promotes the activation and proliferation of fibroblasts, ultimately resulting in an excessive accumulation of extracellular matrix (ECM) proteins due to increased myofibroblast activity (4).

Fibrotic progression in PAP presents significant clinical challenges, as it not only worsens prognosis and reduces treatment responsiveness but also remains poorly understood (5–7). While fibrosis in PAP is thought to result from chronic inflammation, impaired alveolar clearance, and persistent GM-CSF signaling defects leading to dysregulated extracellular matrix deposition, several additional mechanisms have been proposed. One hypothesis suggests that the accumulation of intra-alveolar material disrupts local cytokine signaling, triggering proinflammatory and profibrotic pathways (8). However, it remains unclear why this response is more pronounced in some patients, even with timely diagnosis. Since PAP-PF is a rare complication, even in cases of persistent or residual disease, this suggests that factors beyond disease duration or severity may influence the likelihood of fibrosis progression (9). Identifying PAP patients at risk of fibrosis is critical, as early intervention could help slow disease progression and improve clinical outcomes.

A study involving 44 PAP patients found a relatively high incidence of pulmonary fibrosis (20%), which was linked to a poor prognosis (10). While the imaging and histopathological features of uncomplicated PAP are well-established, radiological and pathological descriptions of PAP-associated pulmonary fibrosis (PAP-PF) remain scarce (11). This gap in knowledge highlights the need for enhanced diagnostic and monitoring tools to better identify and manage PAP patients at risk for fibrotic progression.

Blood biomarkers represent a promising tool for detecting early fibrotic changes in PAP, offering a non-invasive, easily accessible method to monitor disease evolution. Biomarkers associated with alveolar injury, inflammation, and extracellular matrix turnover–such as serum KL-6, SP-D, CCL-18, and matrix metalloproteinases–have been studied in fibrotic lung diseases, including idiopathic pulmonary fibrosis (IPF) and connective tissue disease-associated interstitial lung disease. Their utility in PAP remains an area of active investigation, with potential applications in predicting disease progression, guiding treatment decisions, and tailoring follow-up strategies.

By integrating blood biomarker analysis into routine clinical management, physicians may be able to stratify PAP patients based on their risk of developing fibrosis and implement a more personalized approach to care. High-risk patients could benefit from closer follow-up, earlier therapeutic interventions, and enrollment in clinical trials targeting fibrotic pathways. Additionally, biomarker-guided monitoring could help assess treatment response and refine management strategies over time. A precision medicine approach leveraging blood-based biomarkers could ultimately lead to improved outcomes by facilitating timely, individualized interventions in PAP patients at risk of fibrotic progression.

The ELF (Enhanced Liver Fibrosis) score is a serum biomarker panel that measures three collagen metabolites: type III procollagen amino terminal propeptide (PIIINP), tissue inhibitor of metalloproteinases-1 (TIMP-1), and hyaluronic acid (HA) (12). Originally developed for assessing fibrosis in chronic liver disease, the ELF score has also been explored in pulmonary fibrosis associated with systemic diseases due to the role of these metabolites in extracellular matrix (ECM) remodeling (13–16).

Amino-terminal propeptide of type III procollagen is released during the conversion of type III procollagen to type III collagen and subsequently enters the systemic circulation, making it a recognized marker of collagen synthesis (17). HA, a major non-sulfated glycosaminoglycan in the lung, has been implicated in fibrotic lung diseases, including interstitial pneumonia (18, 19). TIMP-1, a member of the TIMP family, plays a key role in ECM regulation by inhibiting matrix metalloproteinases and has been detected in interstitial cells–primarily macrophages and fibroblast-like cells–in patients with idiopathic pulmonary fibrosis (20).

Given these findings, our study investigates the potential utility of the ELF score as a serum biomarker for pulmonary fibrosis in PAP patients, aiming to improve early detection and disease monitoring.

## 2 Materials and methods

### 2.1 Study population and sample collection

This retrospective study included serum samples from 21 patients diagnosed with autoimmune pulmonary alveolar proteinosis (aPAP), based on clinical presentation, HRCT findings, bronchoalveolar lavage (BAL) cytology and GM-CSF autoantibody positivity (21) who were followed at the Pulmonology Unit of Fondazione IRCCS Policlinico San Matteo, Pavia. Blood samples were collected at the time of a PAP diagnosis and stored at −80 °C until analysis. All patients included in the study had a minimum clinical follow-up of 9 years and provided informed consent prior to participation in the study, which has been approved by the Ethic Committee of Fondazione IRCCS Policlinico San Matteo (Prot. 20210017440).

### 2.2 ELF test and serum biomarker analysis

The Enhanced Liver Fibrosis (ELF) Test™ (Siemens) was used to assess the potential of serum biomarkers as prognostic indicators of pulmonary fibrosis in aPAP. Originally designed for evaluating liver fibrosis, the ELF test was adapted in this study to investigate fibrotic progression in autoimmune PAP. Serum analysis was performed using the ADVIA Centaur XPT system, an automated immunoassay platform that ensures precise and reproducible biomarker quantification. The ELF score derived from these measurements was analyzed as a potential prognostic tool for identifying patients at risk of pulmonary fibrosis progression.

### 2.3 Statistical analysis

Statistical analysis was performed to evaluate differences between the two patient groups. Continuous variables were expressed as mean ± standard deviation (SD). A two-tailed unpaired *t*-test was used to compare serum biomarker levels and ELF scores between autoimmune PAP patients with pulmonary fibrosis (PAP-FIB group) and those without fibrosis (PAP group). To account for differences in pulmonary function tests (PFT), regression models incorporating DLCO were fitted. Receiver operating characteristic (ROC) curve analysis was performed to determine an optimal cut-off value for ELF and its components in distinguishing between the two groups. The area under the ROC curve (AUROC), specificity, and sensitivity were assessed. A *p*-value of less than 0.05 (*p* < 0.05) was considered statistically significant. All analyses were conducted using STATA v18.5.

## 3 Results

### 3.1 Demographic and clinical characteristics

This retrospective study analyzed two groups of autoimmune PAP (aPAP) patients: those who developed pulmonary fibrosis, based on HRCT imaging performed during the follow-up period (22, 23), (PAP-FIB, *n* = 10) and those who did not (PAP, *n* = 11) after at least 9 years of follow-up. There was no significant difference in age at diagnosis between the groups (43 ± 13 vs. 34 ± 12 years, *p* = 0.13). The male-to-female ratio was comparable (6/5 in PAP vs. 8/2 in PAP-FIB). Smoking history did not differ significantly, with a similar proportion of never-smokers and former smokers in both groups (Table 1).

**TABLE 1:** Comparison of demographic and baseline PFT in PAP and PAP-FIB patients.

Demographic or clinical parameter	PAP (*n* = 11)	PAP-FIB (*n* = 10)	*P*-value
**Demographics**			
Age at diagnosis (years)	34 ± 12	43 ± 13	NS
Male/female ratio	6/5	8/2	
Smoking history (never/former/current)	6/4/1	2/5/3	
**Pulmonary function tests (PFTs) and DLCO**			
FVC (% predicted)	78.12 ± 13.67	66.5 ± 17.46	NS
TLC (% predicted)	74 ± 8.08	59.75 ± 14.06	NS
FEV_1_ (% predicted)	77 ± 12.15	65 ± 12.46	NS
FEV_1_/FVC ratio	102.62 ± 8.47	101.6 ± 9.61	NS
DLCO (% predicted)	59.90 ± 19.07	41 ± 12.43	**0.04**
**Disease severity score (DSS)**	2 ± 0.5	3 ± 0.5	0.05

Data are presented as mean ± SD or *n* (%). Statistical significance set at *p* < 0.05 (bold value). NS, not significant.

### 3.2 Pulmonary function tests and DLCO

At aPAP diagnosis, pulmonary function tests revealed a restrictive ventilatory defect with reduced volumes in both PAP and PAP-FIB patients (FVC%: 78.12 ± 13.67 vs. 66.5 ± 17.46; TLC%: 74 ± 8.08 vs. 59.75 ± 14.06). Also FEV_1_ and the FEV_1_/FVC ratio did not differ significantly between the groups FEV_1_%:77 ± 12.15 vs. 65 ± 12.46; FEV_1_/FVC: 102.62 ± 8.47 vs. 101.6 ± 9.61). However, DLCO% was significantly reduced in PAP-FIB patients (59.90 ± 19.07 in PAP vs. in PAP-FIB 41 ± 12.43, *p* = 0.04), consistent with moderately severe lung disease (Table 1).

### 3.3 Disease severity score (DSS)

Participants were given a PAP disease severity score (DSS) according to the presence of symptoms and the extent of PaO2 reduction at diagnosis. As previously described (24), The categories were as follows: DSS 1 = no symptoms and PaO2 ≥ 70 mm Hg; DSS 2 = symptomatic with PaO2 ≥ 70 mm Hg; DSS 3 = PaO2 between 60 mm Hg and 70 mm Hg; DSS 4 = PaO2 between 50 mm Hg and 60 mm Hg; DSS 5 = PaO2 < 50 mm Hg. Qualifying symptoms included dyspnea or cough associated with PAP. In this comparison, PAP-FIB patients have a mean Disease Severity Score (DSS) of 3, while PAP have a mean DSS of 2. The *p*-value of 0.05 indicates a statistically significant difference between the two groups.

### 3.4 ELF score

The ELF score was found to be significantly higher in the PAP-FIB group (9.19 ± 0.75) compared to the PAP group (8.52 ± 0.46), positive score > 7.7), with a significant increase in PAP-FIB group (*p* = 0.02).

**FIGURE 1:**
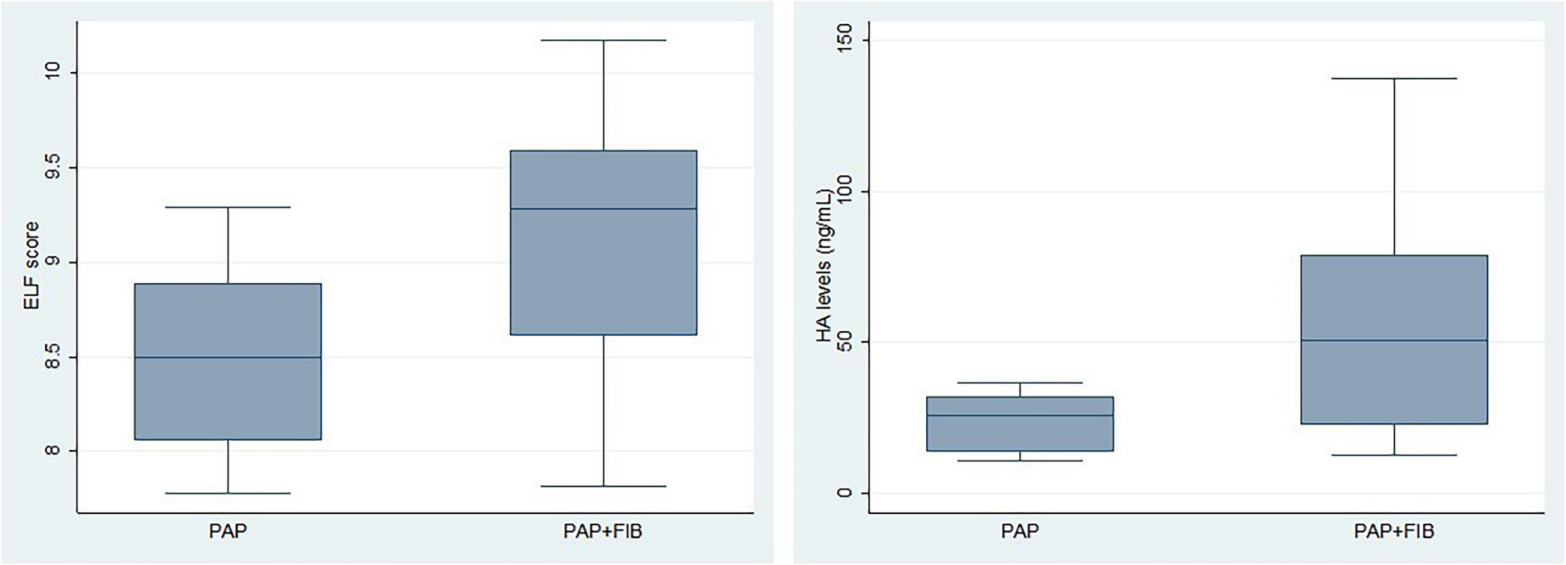
Boxplots illustrating the distribution of ELF scores (left) and HA levels (right) in PAP and PAP-FIB groups. Each box represents the interquartile range (IQR), with the lower and upper edges corresponding to the first (Q1) and third quartiles (Q3), respectively. The horizontal line inside each box indicates the median value, while the whiskers extend to the minimum and maximum values within 1.5 times the IQR. Outliers beyond this range are shown as individual points.

Furthermore, serum HA levels were considerably higher in PAP-FIB patients (55.61 ± 42.01 ng/mL) than in the PAP group (22.83 ± 9.13ng/mL, *p* = 0.02, positive range > 34.6 + 8.8) (Figure 1 and Table 2). The ELF test demonstrated an AUROC of 0.8, with an optimal cut-off value of 9, yielding a sensitivity of 70% and a specificity of 91%. Similarly, HA exhibited an AUROC of 0.8, with a cut-off value of 41 ng/mL, achieving a specificity of 100% and a sensitivity of 60%.

**TABLE 2:** Enhanced Liver Fibrosis (ELF) test.

Enhanced Liver Fibrosis (ELF) test	PAP (*n* = 11)	PAP-FIB (*n* = 10)	*P*-value	DLCO corrected *p*-value
ELF	8.52 ± 0.46	9.19 ± 0.75	**0.02**	NS
TIMP1 (ng/mL)	198.1 ± 213.3	212.84 ± 50.79	NS	
PIIINP (ng/mL)	7.92 ± 1.71	9.24 ± 3.53	NS	
HA (ng/mL)	22.83 ± 9.13	55.61 ± 42.01	**0.02**	**0.05**

Data are presented as mean ± SD or *n* (%). Statistical significance set at *p* < 0.05 (bold values). NS, not significant.

## 4 Discussion

This retrospective study aimed to assess the potential of serum biomarkers, specifically the ELF Test™ (Siemens), as prognostic indicators of pulmonary fibrosis in autoimmune pulmonary alveolar proteinosis (aPAP). The ELF test, originally designed for liver fibrosis, was adapted to evaluate fibrotic progression in aPAP patients.

The study’s two patient cohorts, those with pulmonary fibrosis (PAP-FIB) and those without fibrosis (PAP), were well-matched in terms of age at diagnosis, sex distribution, and smoking history, indicating that these factors were unlikely to contribute significantly to differences in disease progression. However, while at the diagnosis both groups exhibited restrictive ventilatory defects, patients in the PAP-FIB group had lower lung function values than those in the PAP group, as indicated by lower forced vital capacity (FVC%) and total lung capacity (TLC%). The most striking difference was observed in DLCO%, which was significantly reduced in PAP-FIB patients (*p* = 0.04). This data suggests that patients who will develop fibrosis may already have an epithelial or endothelial alteration at the time of diagnosis, which could subsequently drive the development of pulmonary fibrosis. The role of DLCO as a prognostic factor in PAP has been evaluated in several studies (25, 26) and appears to be correlated with the development of cardiovascular diseases in these patients (27). Further analyses are needed to determine whether the percentage reduction in predicted DLCO could serve as a predictive factor for fibrosis development in these patients.

The lack of significant differences in FEV_1_ and FEV_1_/FVC ratios between the groups may indicate the inadequacy of dynamic volumes to identify differences between the two groups and that other factors beyond basic lung function measures may contribute to the progression of fibrosis in aPAP.

Disease severity, as measured by the PAP Disease Severity Score (DSS), was also significantly higher in PAP-FIB patients (mean DSS of 3) compared to non-fibrotic PAP patients (mean DSS of 2, *p* = 0.05). This finding aligns with the observed decline in pulmonary function and suggests that more severe initial disease presentation may predispose patients to fibrotic progression over time.

The findings of this study underscore the potential prognostic value of the ELF test and its individual components, particularly hyaluronic acid (HA), in assessing pulmonary fibrosis in patients with autoimmune PAP. Given that an ELF score above 7.7 is considered indicative of fibrotic activity, this result suggests that the ELF test, despite its original use in liver fibrosis, may have applicability in detecting pulmonary fibrosis risk in aPAP. The increased ELF score in PAP-FIB patients reflects enhanced extracellular matrix remodeling and fibrotic processes, reinforcing the biomarker’s potential role in risk stratification.

Interestingly, both groups exhibited mean ELF scores above the prognostic cutoff of 7.7, indicating that even non-fibrotic aPAP patients may have underlying fibrotic activity or a predisposition toward fibrosis. This finding suggests that ELF scores alone may not be sufficient to distinguish between those who will progress to fibrosis and those who will not.

The markedly higher serum HA levels in the PAP-FIB group further highlight its role as a potential biomarker for PAP-associated fibrosis. HA is a major ECM component involved in tissue remodeling, inflammation, and fibrogenesis (28). Its increase in fibrotic patients suggests an association with fibroblast activation and the imbalance between ECM synthesis and degradation. Increased HA levels are also reported in the literature to be associated with the development of epithelial-mesenchymal transition (EMT), which may suggest a role in pulmonary fibrosis progression (29).

The presence of persistently high HA levels in PAP-FIB patients implies ongoing tissue injury and chronic inflammatory responses that may contribute to progressive lung fibrosis.

Importantly, when applying a regression model accounting for differences in DLCO, the difference in ELF scores between the two groups was no longer statistically significant. This suggests that ELF score differences may be largely driven by pulmonary function impairment rather than acting as an independent predictor of fibrosis. However, the difference in HA levels remained marginally significant (*p* = 0.05), indicating that HA may serve as a more robust independent biomarker for fibrotic progression in aPAP. This finding emphasizes the need for further research to clarify the relative contributions of different biomarkers and pulmonary function metrics in predicting fibrosis in aPAP patients.

Furthermore, the diagnostic performance of ELF and HA as predictive biomarkers was evaluated using receiver operating characteristic (ROC) analysis. The ELF score demonstrated an area under the ROC curve (AUROC) of 0.8, with an optimal cut-off value of 9, providing a sensitivity of 70% and specificity of 91%. Similarly, HA also exhibited an AUROC of 0.8, but with a cut-off of 41 ng/mL, it achieved a specificity of 100% and a sensitivity of 60%. These findings suggest that while the ELF score provides a balanced predictive value, HA, despite its lower sensitivity, may serve as a highly specific marker for identifying patients at risk of fibrosis. The combination of these biomarkers could enhance the accuracy of prognostic assessments, warranting further validation in larger cohorts.

These findings support the hypothesis that HA could serve as an early, non-invasive biomarker for detecting fibrotic changes in aPAP patients. Regular monitoring of HA levels may facilitate early identification of individuals at risk of fibrosis, allowing for more targeted follow-up and timely therapeutic interventions aimed at mitigating irreversible lung damage. Furthermore, the ability of the ELF score to differentiate fibrotic from non-fibrotic aPAP patients suggests its potential utility in clinical practice as part of a composite assessment alongside functional parameters for risk stratification and disease progression monitoring.

Despite these promising results, several limitations should be acknowledged. The relatively small sample size of this study may limit the generalizability of the findings, necessitating validation in larger, multi-center cohorts. Additionally, the mechanisms underlying HA elevation in aPAP-related fibrosis require further investigation to elucidate its precise role in disease pathogenesis. Future studies should also explore whether changes in HA levels over time correlate with treatment response and long-term clinical outcomes in PAP patients.

In conclusion, this study provides compelling evidence for the role of ECM biomarkers, particularly HA, in the pathophysiology of PAP-associated fibrosis. The significantly higher ELF scores and HA levels in fibrotic patients highlight the importance of ECM remodeling in disease progression. Incorporating biomarker analysis into routine clinical management may enhance risk stratification, facilitate early intervention, and improve overall patient outcomes. Further studies with larger cohorts and longitudinal data are warranted to refine these findings and establish standardized protocols for biomarker-driven monitoring and therapeutic strategies in PAP patients.

## Data Availability

The original contributions presented in this study are included in this article/supplementary material, further inquiries can be directed to the corresponding author.
